# Role of Doppler Waveforms in Pregnancy-Induced Hypertension and Its Correlation With Perinatal Outcome

**DOI:** 10.7759/cureus.18888

**Published:** 2021-10-19

**Authors:** Ranjumoni Konwar, Bharati Basumatari, Malamoni Dutta, Putul Mahanta, Ankumoni Saikia, Rashmi UK

**Affiliations:** 1 Radiology, Fakhruddin Ali Ahmed Medical College and Hospital, Barpeta, IND; 2 Anatomy, Assam Medical College, Dibrugarh, IND; 3 Forensic Medicine and Toxicology, Assam Medical College and Hospital, Dibrugarh, IND; 4 Community Medicine, Gauhati Medical College and Hospital, Guwahati, IND; 5 Radiology, Gauhati Medical College and Hospital, Guwahati, IND

**Keywords:** perinatal outcome, cerebro-placental ratio, middle cerebral artery, umbilical artery, pregnancy-induced hypertension

## Abstract

Objectives

The present study aims to measure the role of Doppler waveforms in pregnancy-induced hypertension (PIH) and its relationship with the perinatal outcome.

Methods

We have studied 50 pregnant women with PIH with gestational age (GA) 30-40 weeks for Umbilical Artery (UmA), Middle Cerebral Artery (MCA) and Uterine Artery (UtA) Doppler waveforms. Comparison between the various Doppler indices, i.e., Pulsatility Index (PI), Resistive Index (RI) and S/D ratio, with the severity of the disease and the perinatal outcomes were evaluated using appropriate statistical tests considering a threshold value of p-value <0.05 as significant. The Statistical Package for the Social Sciences (SPSS) version-16 (SPSS Inc, Chicago, USA) and MedCalc software (MedCalc Software Ltd, Ostend, Belgium) were used for data analysis.

Results

Half (50%) of the cases attributed to the 26 to 30 years age group were at 38 to 40 weeks of gestation. Out of the 50 patients, 68% were primigravida, and 74% had severe PIH. Mean UmA PI, mean MCA PI, mean MCA RI, and mean Cerebro Placental Ratio (CPR) were differed significantly among mild and severe PIH patients (p-value<0.05). Perinatal outcomes in 33 (66%) cases were adverse. The abnormal UmA RI, MCA RI, MCA PI, MCA S/D were significantly linked with poor pregnancy outcomes (p-value <0.05). PIH cases with the presence of early diastolic notch of UtA (p-value <0.01), abnormal PI CPR (p-value <0.001) and S/D CPR (p-value <0.003) were observed to have more adverse outcomes. PI CPR had the highest sensitivity (84.8%), and the existence of early diastolic notch of UtA and MCA-PI were most specific in diagnosing adverse perinatal outcomes.

Conclusion

CPR-PI is a valuable indicator of adverse perinatal outcomes in PIH. Doppler studies of multiple vessels may help manage high-risk pregnancies as it may provide helpful information about the fetus at risk of hypoxia and placental insufficiency.

## Introduction

Hypertensive disorders affect approximately 5-10% of all pregnancies worldwide [1]. It is the most typical medical condition in pregnancy. As a significant risk factor of maternal and perinatal mortality and morbidity globally, it accounts for almost 10-20% of pregnancy-related mortality in low and middle-income countries [2]. Hypertension during pregnancy results in uteroplacental insufficiency. It is considered a major contributing factor in adverse post-delivery outcomes such as newborn intensive care unit (NICU) admission, low birth weight, birth asphyxia, preterm birth, perinatal death, intrauterine growth restriction and stillbirth [3].

As per the classification recommended by the National High Blood Pressure Education Program Working Group on High Blood Pressure in Pregnancy, hypertensive disorders in pregnancy are classified as chronic hypertension, preeclampsia-eclampsia, pre-eclampsia superimposed on chronic hypertension, and gestational hypertension [4].

Doppler ultrasound, based on the physical principle first described by CA Doppler in 1842, states the change in frequency of a sound wave when a moving object reflects it provides a safe, non-invasive, and rapid method to assess uteroplacental and fetal circulation, which helps in examining the relationship of impaired blood flow to adverse perinatal outcome [5,6].

In this study, we investigated the role of Doppler Ultrasound of Umbilical Artery (UmA), Middle Cerebral Artery (MCA) and Uterine Artery (UtA) in foretelling adverse perinatal outcomes in pregnancies complicated by pregnancy-induced hypertension (PIH) and determining the role of Doppler Velocimetry in the clinical management of such pregnancy.

This study aims to assess the role of Doppler waveforms in PIH and its correlation with the perinatal outcome among pregnancies with PIH admitted to a tertiary care center in the northeastern region of India.

## Materials and methods

A prospective study was conducted among 50 pregnant women with PIH in the Department of Obstetrics and Gynaecology, collaborating with the Department of Radiology, Gauhati Medical College and Hospital, Guwahati. Singleton pregnancies with 30 to 40 weeks gestational age (GA), complicated by PIH, attending antenatal Out-Patient Department (OPD) and labour room are included. The study excluded pregnancies with a significant congenital abnormality, multiple gestations, and intrauterine death at the first Doppler examination. Sixty-one patients meeting the inclusion and exclusion criteria were initially selected using a convenient sampling method during the one-year study period. However, 11 participants were further excluded due to follow-up loss (seven cases) and incomplete data (four patients).

Before the Doppler Ultrasound evaluations, a detailed clinical history, ultrasound biometry, amniotic fluid assessment, and placental maturity were conducted. Follow-up Doppler studies were performed for identifying any specific trend in the Doppler indices. The last Doppler ultrasound results were used to analyse the perinatal outcomes.

After ultrasound biometry assessment, the pregnant women were subjected to Doppler studies of the UmA, MCA and UtA serially between 30-40 weeks. An ultrasound machine, Siemens, Acuson Antares premier edition (Siemens AG, Munich, Germany), and probe of 2-5 MHz with high pass filter were used to perform the Doppler assessments. Doppler indices were obtained by plotting the measurements graphically following normograms provided by Harrington et al., [7]. We acquired the waveforms during fetal inactivity and apnea. UmA Doppler flow velocity waveforms were taken from a free loop of the cord equidistant from the placenta and abdominal wall insertion. Measurements were recorded when a clear waveform was attained, not including fetal breathing or body movement. For MCA-Doppler waveforms, the fetal head was transversely imaged at the level of the sphenoid bones, and the circle of Willis was displayed by colour flow imaging. The MCA in the near field was insinuated about 1 cm distal to its origin from the internal carotid artery. In both cases, the angle of insinuation was <60º. UtA was positioned at a point just distal to the crossover with the iliac artery before the uterine artery divides into arcuate arteries.

We also noted the flow velocity waveforms of UmA, MCA and UtA, where S is the maximum peak systolic frequency, D is the end-diastolic, and A is the mean Doppler shift frequency during a cardiac cycle. Results of the Doppler examination were noted, and follow-up Doppler studies are done as and when required.

Doppler ultrasound values were examined for the prediction of adverse perinatal outcomes, which includes low birth weight (below 10th percentile), perinatal death comprising intrauterine death and early neonatal death, emergency caesarean section due to fetal distress, Low Apgar score of below 7 (at 5 minute) and admission to NICU. The pregnancy outcome was uneventful or favourable in the absence of the above conditions.

The UmA-Pulsatility Index (PI) ratios above the 95th percentile and MCA-PI value below the 5th percentile of previously published values for GA were deemed abnormal. The MCA/UmA PI ratio, i.e., Cerebro Placental Ratio (CPR)-PI, was considered abnormal when less than 1.08 [8].

Statistical analysis

Descriptive statistics were presented as frequencies and proportions. The difference in means was tested using the t-test or Mann-Whitney U test. The Chi-square test was used to detect the associations between categorical variables. Diagnostic accuracies for all Doppler measurements were evaluated using sensitivity analysis. The data were analysed by Statistical Package for the Social Sciences (SPSS) version-16 (SPSS Inc., Chicago, USA) and MedCalc software (MedCalc Software Ltd, Ostend, Belgium). We took the ethical clearance from the ethics committee of Gauhati Medical College, Guwahati, Assam and took the informed consent from the included participants.

## Results

A prospective study was carried out, including 50 antenatal patients satisfying the inclusion and exclusion criteria. Flow velocity waveforms from all the study participants were analysed.

The age range of the patients was from 21 to 33 years, and half of them belonged to the age group 26 to 30 years (50%). The mean±standard deviation (s.d.) age of the patients was 26.6±3.1 years. Most of the patients (70%) were between 38 to 40 weeks of gestation at Doppler examination. About 68% of the studied women were primigravida, and 74% were nulliparous. Out of 50 cases, nine (18%) had a previous history of abortion. Among the 50 PIH cases, 74% of patients had severe PIH, as shown in Table 1.

**Table 1:** Demographic and clinical profile of the pregnancy-induced hypertension cases

Characteristics	Number (n=50)	Percent
Age group		
<=25 years	20	40.0
26-30 years	25	50.0
31-35 years	5	10.0
Gestational age (GA)		
30 -34 weeks	3	6
> 34 -37 weeks	12	24
>37-40 weeks	35	70
Severity of Hypertension		
Mild (≥140/90)	13	26
Severe (≥160/110)	37	74
Obstetric history		
Primigravida	34	68%
Parity 0	37	74%
Previous history of abortion	9	18%

As seen from Table 2, the majority (70%) of the deliveries were spontaneous and at term. A total of 34 (68%) deliveries had adverse outcomes, of which 19 had multiple adverse effects.

**Table 2:** Maternal outcomes of the 50 pregnancies

Maternal outcome	Number (n=50)	Percent
Mode of delivery		
Spontaneous vaginal delivery	35	70%
Lower segment Caesarean section (LSCS)	15	30%
Duration of gestation at termination		
Term	35	70%
Preterm	15	30%
Pregnancy outcome		
Uneventful	16	32%
Adverse	34	68%

**Figure 1:**
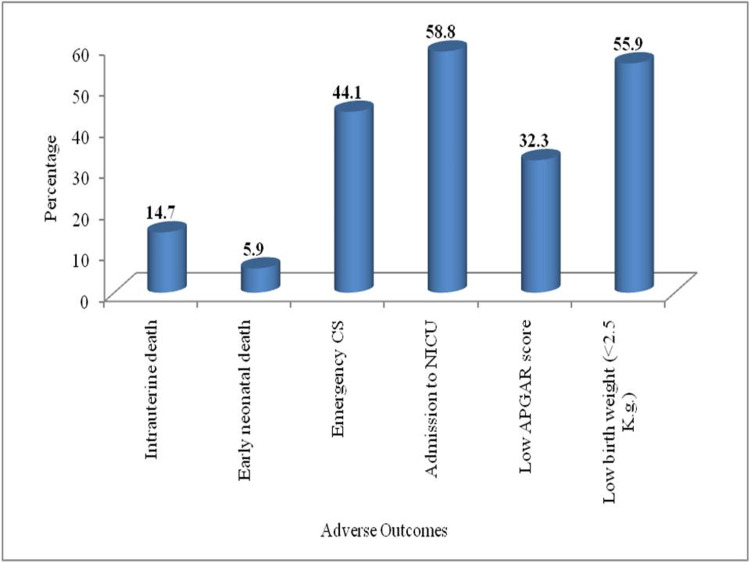
Distribution of adverse outcomes among the pregnancy-induced hypertension cases

Newborn intensive care unit (NICU) admissions (58.8%), low birth weight (55.9%) and emergency caesarean section (44.1%) were the highest encountered adverse outcomes (Figure 1).

Mean UmA-PI was considerably higher in severe PIH cases than mild cases (p-value <0.05). Means of both MCA-Resistive Index (RI) and MCA-PI values differed among mild and severe PIH patients highly significantly and are lower among severe PIH cases (p-value <0.01). Also, the mean CPR among the mild PIH group was substantially higher (p-value <0.01) than the severe PIH cases, as shown in Table 3.

**Table 3:** Doppler parameters and severity of pregnancy-induced hypertension UmA: Umbilical Artery; UtA: Uterine Artery; MCA: Middle Cerebral Artery; CPR: Cerebro Placental Ratio; RI: Resistive Index; PI: Pulsatility Index; S/D: systolic/diastolic ratio; PIH: pregnancy-induced hypertension

Doppler parameters	Mild PIH group	Severe PIH group	p-value
UmA RI	0.59±0.57	0.66±0.57	0.21
UmA PI	0.98±0.94	1.18±1.21	0.02
UmA S/D	2.70±2.71	2.98±2.88	0.20
UtA RI	0.62±0.55	0.67±0.71	0.07
UtA PI	1.58±1.44	1.66±1.68	0.14
MCA RI	0.71±0.73	0.65±0.66	0.009
MCA PI	1.30±1.32	1.15±1.13	0.009
MCA S/D	3.91±4.21	3.63±3.76	0.07
CPR	1.34±1.4	1.02±0.94	0.003

The Chi-square test was performed to test the association between Doppler features and pregnancy outcomes among PIH cases. The RI abnormality of UmA was found significantly related to unfavourable pregnancy outcomes (p-value <0.05). Similarly, an early diastolic notch of UtA was found to influence the perinatal outcome substantially. Perinatal results were also found to be linked with various MCA Doppler indices, and it is observed that adverse effects significantly encountered by the PIH patients who had abnormal RI (p-value <0.05), abnormal PI (p-value <0.01) and abnormal systolic/diastolic (S/D) ratio (p-value <0.01) of MCA. PIH patients with abnormal CPR-PI (p-value <0.001) and CPR S/D (p-value =0.003) were observed to have more adverse outcomes compared to those having normal CPR, as shown in Table 4.

**Table 4:** Association of Doppler measurements with the perinatal outcome UmA: Umbilical Artery; UtA: Uterine Artery; MCA: Middle Cerebral Artery; CPR: Cerebro Placental Ratio; RI: Resistive Index; PI: Pulsatility Index; S/D: systolic/diastolic ratio

Doppler Measurements	Result	Adverse outcome	Uneventful Outcome	p-value
UmA RI	Abnormal	20	5	0.04
	Normal	13	12	
UmA PI	Abnormal	20	7	0.19
	Normal	13	10	
UmA S/D	Abnormal	25	10	0.22
	Normal	8	7	
UtA RI	Abnormal	25	12	0.69
	Normal	8	5	
UtA PI	Abnormal	20	8	0.36
	Normal	13	9	
UtA S/D	Abnormal	23	8	0.11
	Normal	10	9	
Early Diastolic notch of UtA	Present	20	3	0.004
	Absent	13	14	
MCA RI	Abnormal	22	5	0.01
	Normal	11	12	
MCA PI	Abnormal	22	3	0.001
	Normal	11	14	
MCA S/D	Abnormal	27	6	0.001
	Normal	6	11	
CPR (MCA PI/ UmA PI)	Abnormal	28	4	<0.001
	Normal	5	13	
CPR (MCA/ UmA S/D)	Abnormal	22	4	0.003
	Normal	11	13	

As seen from Table 5, CPR of PI had the highest sensitivity (84.8%) and accuracy (82.0%), followed by the S/D ratio of fetal MCA (sensitivity=81.8%, accuracy=76.0%). In the present study, the early Diastolic notch of UtA and MCA PI was most specific in diagnosing adverse perinatal outcomes. The sensitivity of the UtA Doppler study to detect adverse perinatal effects was 72.17% when four Doppler parameters were considered. Among the UmA Doppler parameters, the S/D ratio included the highest sensitivity of 75.8% for predicting adverse outcomes.

**Table 5:** Diagnostic accuracy of Doppler indices in predicting perinatal outcome UmA: Umbilical Artery; UtA: Uterine Artery; MCA: Middle Cerebral Artery; CPR: Cerebro Placental Ratio; RI: Resistive Index; PI: Pulsatility Index; S/D: systolic/diastolic ratio; PPV: positive predictive value; NPV: negative predictive value.

Doppler Measurements	Sensitivity	Specificity	PPV	NPV	Accuracy
UmA RI	60.6	70.6	80.0	48.0	64.0
UmA PI	60.6	58.8	74.1	43.5	60.0
UmA S/D	75.8	41.2	71.4	46.7	64.0
UtA RI	75.8	29.4	67.6	38.5	60.0
UtA PI	60.6	52.9	71.4	40.9	58.0
UtA S/D	69.7	52.9	74.2	47.7	64.0
Early Diastolic notch of UtA	60.6	82.3	87.0	51.8	68.0
MCA RI	66.7	70.6	81.5	52.2	68.0
MCA PI	66.7	82.3	88.0	56.0	72.0
MCA S/D	81.8	64.7	81.8	64.7	76.0
CPR (MCA PI/ UmA PI)	84.8	76.5	87.5	72.2	82.0
CPR (MCA/ UmA S/D)	66.7	76.5	84.6	54.2	70.0

**Figure 2:**
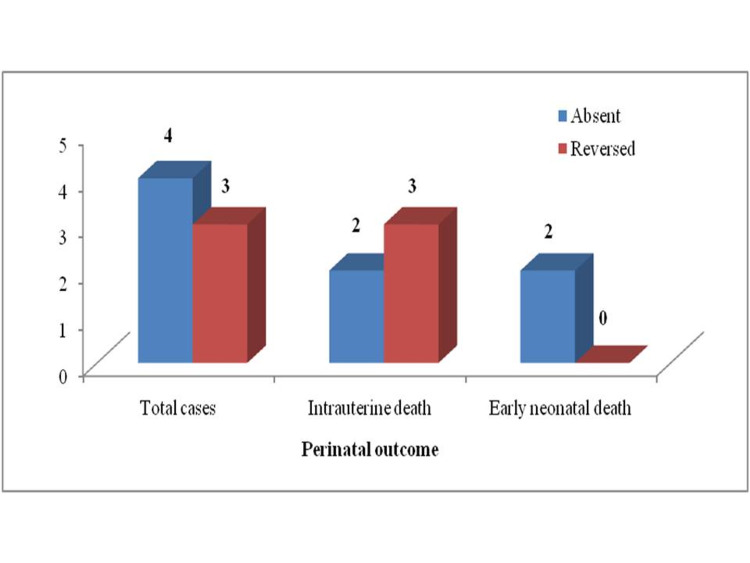
Perinatal mortality versus the end-diastolic flow of the umbilical artery

The perinatal outcome was poor with the absent and reversed UmA diastolic flow with the perinatal mortality being 100%, thus stressing the importance of appropriate, timely intervention in compromised fetuses (Figure 2).

## Discussion

Doppler ultrasound provides a safe and non-invasive method to assess fetal and maternal circulation patterns during pregnancy. The exercise of Doppler ultrasound in PIH specifically in predicting pre-eclampsia and the resulting adverse outcomes was suggested by various studies [9,10]. Doppler velocimetry evaluates abnormal fetal hemodynamics resulting from changes in placental resistance. Doppler indices can help identify foetuses with increased placental and decreased cerebral resistance.

Among the 50 PIH cases, the majority (50%) belonged to the age group 26-30 years. Also, PIH was more frequent among first-time mothers, i.e., primigravida (68%) and nulliparous (74%) women. The findings are concordant to a recent study from Odisha suggesting young expectant mothers are at elevated risk of PIH [11].

The mean birth weight was 2.1 kg±0.26 kg, and 62% of the neonates (n=31) had a birth weight >2.5 kilograms. Out of the 50 high-risk pregnancies, 33 (66%) encountered adverse outcomes with five intrauterine deaths, two early postnatal death, 20 NICU admission, 10 with 5-minute Apgar score <7, 19 had low birth weight, and 15 cases required emergency LSCS.

It is observed that mean UmA-PI was significantly elevated among cases with severe PIH than mild cases (p-value <0.05). Also, both RI and PI values of fetal MCA were substantially lower among severe PIH patients (p-value<0.01). The mean CPR in the mild PIH group was significantly higher (p-value <0.01) than in severe PIH cases. Studies on pre-eclampsia and gestational hypertension patients also observed similar variability in Doppler indices with the severity of the disease [12-14].

The umbilical artery represents the fetoplacental system which primarily reflects placental resistance and is the primary vessel for monitoring high-risk pregnancies. Various studies reported abnormal umbilical artery PI and S/D ratio as factors influencing adverse pregnancy outcomes like intrauterine growth restriction (IUGR), neonatal death and low Apgar score [15,16]. In the current study, adverse outcomes were observed significantly more (p-value =0.04) among PIH cases with abnormal UmA-RI. The early diastolic notch in UtA was also significantly related to unfavourable effects (p-value =0.004). Park et al. also suggested the consequence of notch depth in the presence of diastolic notch to predict poor perinatal outcomes [17]. Also, it is observed that adverse outcomes were substantially higher amongst cases with abnormal fetal MCA measurements and abnormal CPR (p-value <0.05). Similar findings were observed in other studies [18,19].

In the present study, CPR (MCA/UmA PI) had the highest sensitivity (84.8%), positive predictive value (PPV) (87.5%) and accuracy (82.0%) in detecting adverse perinatal outcomes. The sensitivity of PI of MCA/UmA over the other Doppler indices is comparable to various other studies [8, 20-22]. Also, in our research, PI of MCA had the highest specificity in predicting adverse outcomes, which is not correlating with the survey by Smitha et al. [21].

Limitation

The present study included pregnant women attending only one tertiary care centre and thus represents a sample from a single geographic reason. However, as studies on the use of Doppler waveforms in the management of high-risk pregnancies are limited in this part of India, the observations of the present study may add useful information in further research on the topic in a generalised population of northeast India constituting different tribes and ethnicity.

## Conclusions

The Doppler technology is helpful in repetitive noninvasive haemodynamic monitoring of pregnancy. In an obstetric patient population with a high prevalence of complications like PIH, the Doppler indices from the fetal circulation can reliably predict adverse perinatal outcomes.

The cerebroplacental ratio is a valuable predictor of adverse perinatal outcomes. Absent end-diastolic flow and reversal of the umbilical artery flow are ominous findings with a high perinatal mortality rate. Thus the appropriate timing of delivery is to be made before these changes occur. However, the Doppler study of multiple vessels helps predict adverse outcomes and manage high-risk pregnancies complicated by PIH.
